# Case Report: “Methicillin-Resistant *Staphylococcus aureus* Endocarditis Overlying Calcified Mitral Annular Abscess Misdiagnosed as *Klebsiella pneumoniae* Endocarditis”

**DOI:** 10.3389/fmicb.2021.818219

**Published:** 2022-01-18

**Authors:** Hiroyuki Yamamoto, Katsuya Hashimoto, Hiroyuki Yamada, Yoshihiko Ikeda, Takashi Takahashi, Toru Hashimoto

**Affiliations:** ^1^Department of Cardiovascular Medicine, Narita-Tomisato Tokushukai Hospital, Chiba, Japan; ^2^Department of Cardiovascular Surgery, Narita-Tomisato Tokushukai Hospital, Chiba, Japan; ^3^Department of Pathology, National Cerebral and Cardiovascular Center, Suita, Japan; ^4^Laboratory of Infectious Diseases, Graduate School of Infection Control Sciences & Omura Satoshi Memorial Institute, Kitasato University, Tokyo, Japan

**Keywords:** MAC, infective endocarditis, dual bacteremia, Duke criteria, mitral annular abscess, MRSA, TEE

## Abstract

Infective endocarditis (IE) involving mitral annular calcification (MAC) is a rare disease, but is potentially lethal due to frequent serious periannular complications, and therefore requires early diagnosis and prompt treatment. However, either reaching the correct diagnosis or the detection of periannular complications, even with conventional transesophageal echocardiography (TEE), remains challenging because calcium deposition obscures clear visualization of the area around the MAC. We describe a unique case of methicillin-resistant *Staphylococcus aureus* (MRSA) IE involving a calcified mitral annular abscess, which was initially misdiagnosed as *Klebsiella pneumoniae* IE. Accurate diagnosis of MAC-related IE as well as detection of the annular abscess were made possible by 4D TEE, leading to successful cardiac surgery, which confirmed MRSA IE pathologically, and the associated annular abscess. This case highlights the usefulness of 4D TEE for the accurate diagnosis and proper surgical planning. In addition, this case raises the limitations of the modified Duke criteria in cases of definite IE with dual bacteremia.

## Introduction

Infective endocarditis (IE) originating from mitral annular calcification (MAC) is rare but is strongly associated with fatal periannular complications if left untreated (Eicher et al., 2004; Hill et al., 2007). However, various artifacts caused by calcium deposition obscure clear visualization of the MAC region even with conventional transesophageal echocardiography (TEE) (Hill et al., 2007). Therefore, accurate diagnosis and early detection of periannular complications remain exceptionally challenging.

## Case Description

**Figure 1:**
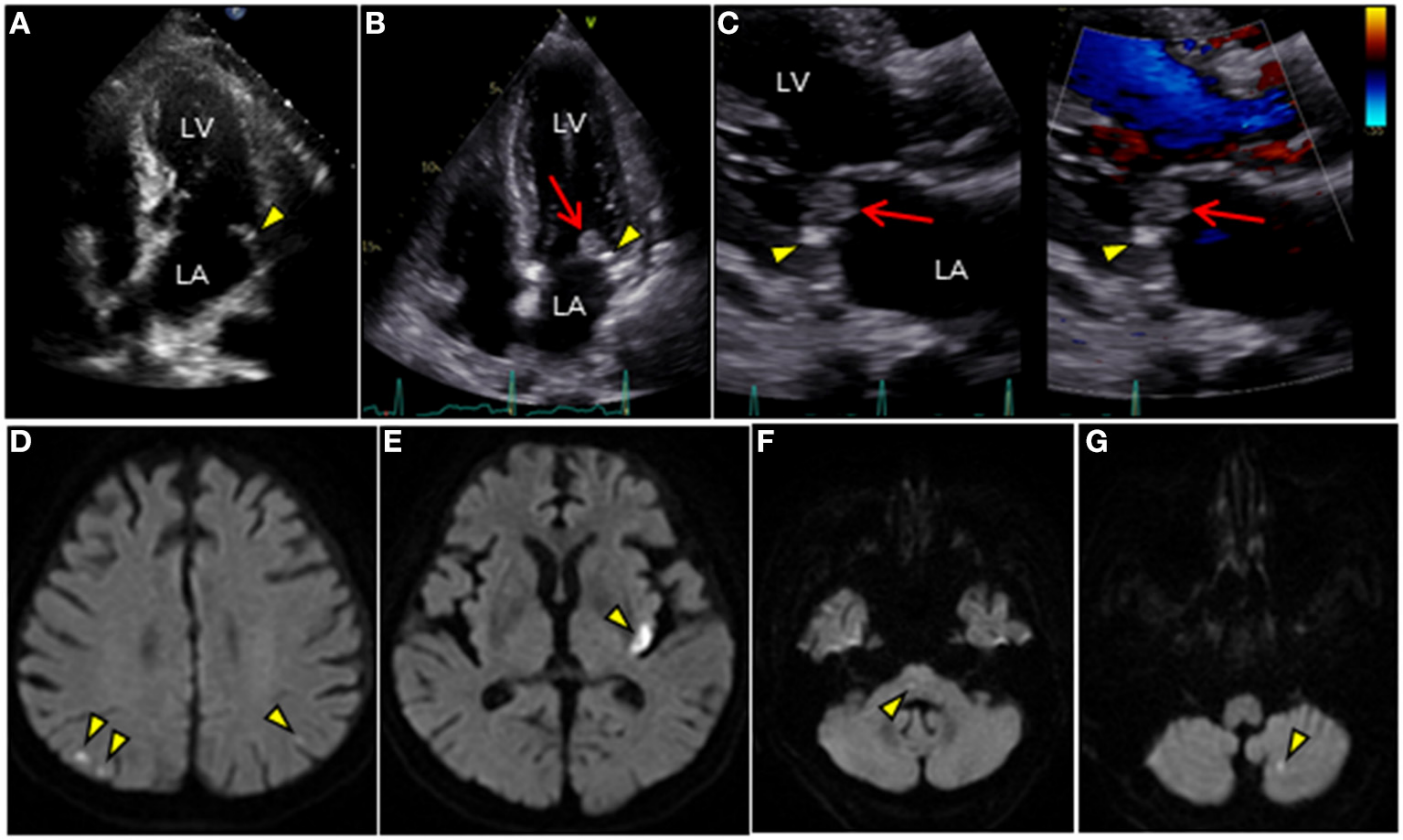
TTE and brain MRI. **(A–C)** Longitudinal TTE: **(A,B)** apical 4-chamber and **(C)** parasternal long-axis views. **(A)** TTE shows tiny MAC along the posterior mitral annulus 1 year ago (arrowhead). **(B,C)** TTE after admission shows MAC progression (arrowhead) and a large round mass superimposed on the MAC (arrow). Mass with wide attachment (10 ×11 mm in size) is characterized by heterogeneity and oscillation. **(C)** Notably, color Doppler TTE showed no significant mitral regurgitation. **(D–G)** Brain diffusion-weighted MRI detects multiple acute cerebral infarctions in both hemispheres (arrowheads). LA, left atrium; LV, left ventricle; MAC, mitral annular calcification; MRI, magnetic resonance imaging; TTE, transthoracic echocardiography.

**Figure 2:**
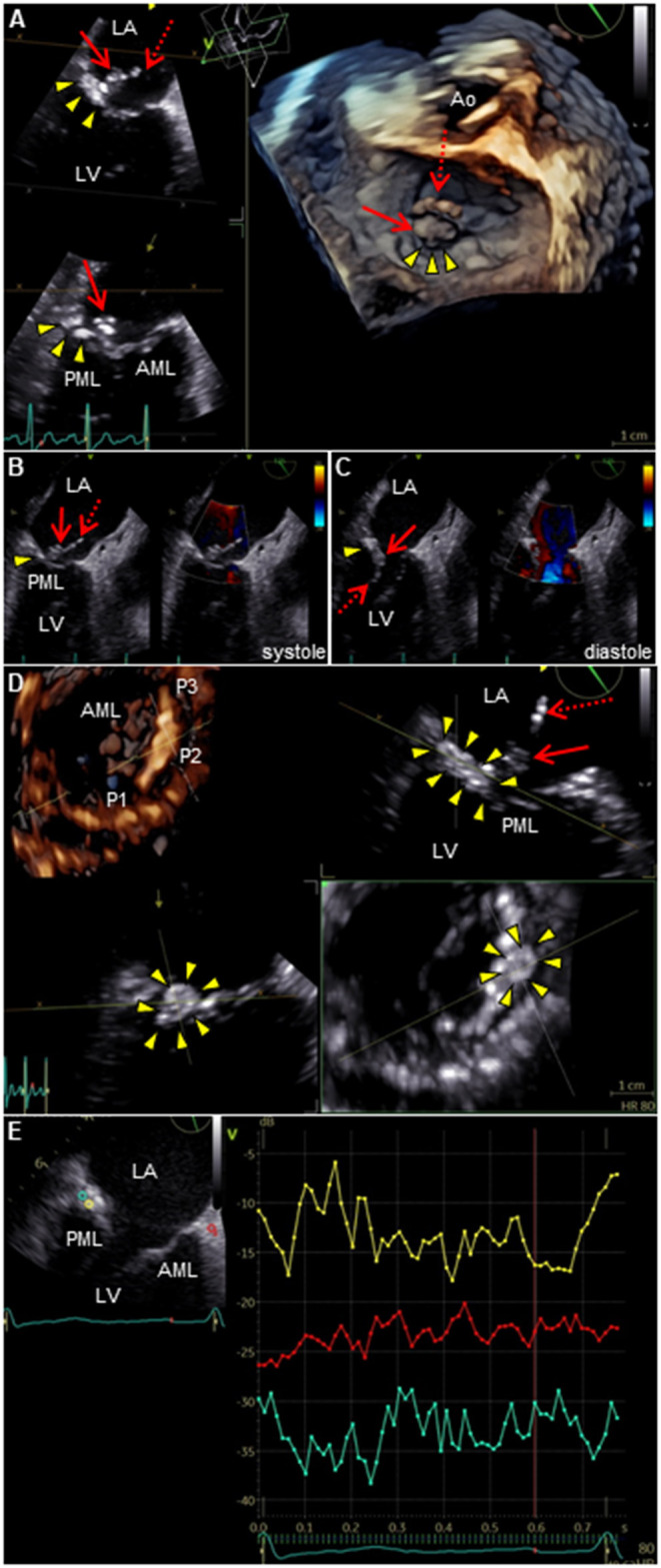
TEE of the mitral valve. **(A)** 3D TEE or **(B,C)** 2D color Doppler TEE shows a long mobile mass of vegetations which consist of body part (arrow) and tail part (dotted arrow), containing calcium-dense stippling, attached to the calcified posterior mitral annulus (arrowheads). Note the intact valve leaflets. **(D)** 4D TEE with Flexi-slice mode demonstrates MAC with a central echolucent area (arrowheads) around the P2 segment of the mitral valve. **(E)** Q-analysis presents a contrast analysis that traces the grayscale intensity in a defined region of interest as a function of time: green, yellow, and red depicts the center of MAC, the periphery of MAC, and normal cardiac muscle tissue as a control, respectively (left). Time-intensity curves are plotted for the respective region of interest (right): X axis, Time (s); and Y axis, Intensity scale (logarithmic) (dB). AML, anterior mitral leaflet; Ao, aorta; LA, left atrium; LV, left ventricle; MAC, mitral annular calcification; PML, posterior mitral leaflet; TEE, transesophageal echocardiography.

**Figure 3:**
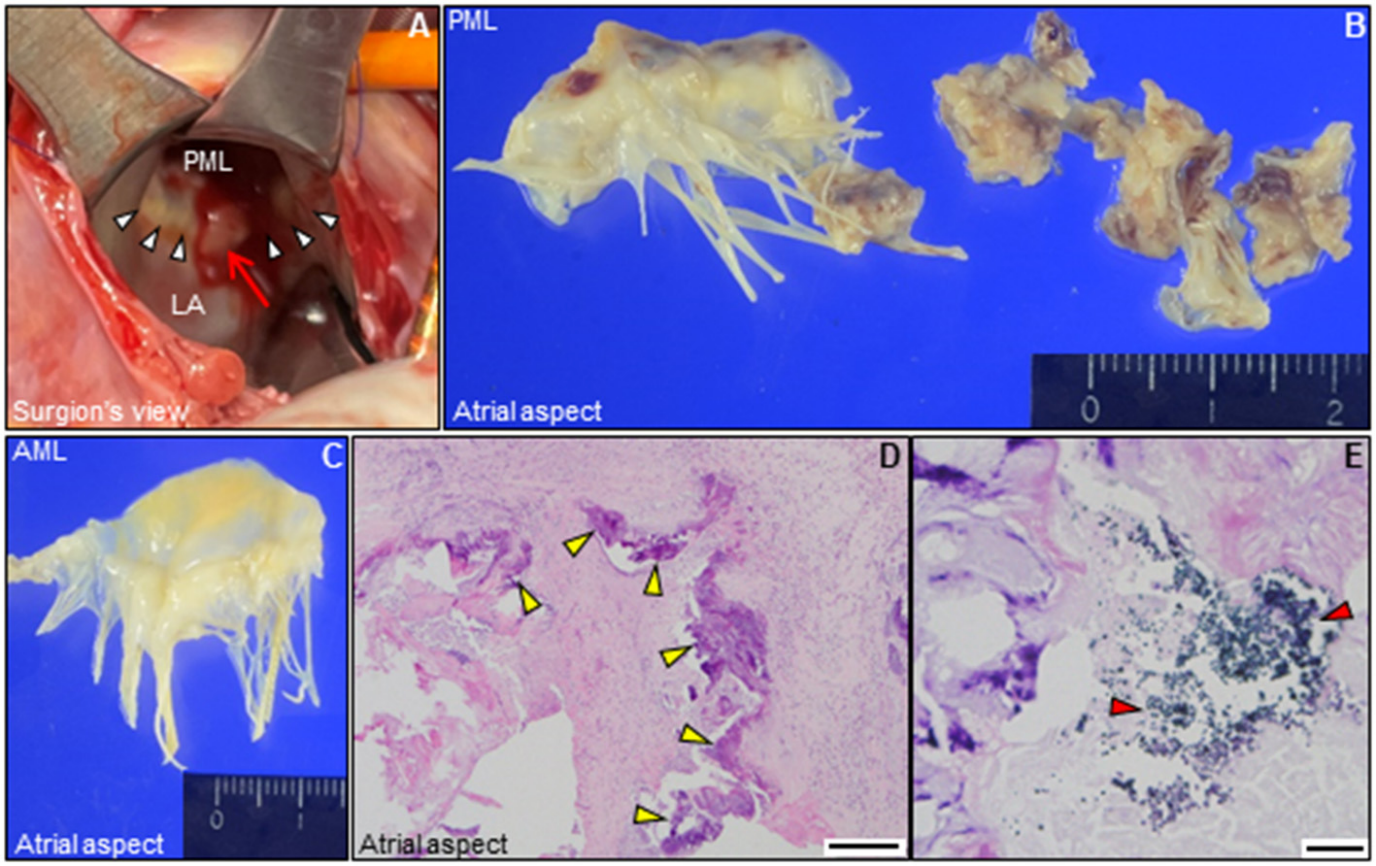
Intraoperative and pathological findings. **(A)** Intraoperative view of the mitral valve. A large mass of vegetations superimposed on mitral annular calcification (arrow) is observed around the P2 segment in the posterior mitral annulus (white arrowheads). **(B,C)** Representative macro-photographs of the extracted mitral valve leaflets (**B**, PML; **C**, AML). **(D,E)** Histological findings of the resected PML shows dystrophic calcification (yellow arrowheads), and surrounding neutrophilic inflammatory cell infiltration and bacterial agglomeration (red arrowheads) [**(D)** hematoxylin and eosin staining, Bar 200 μm; **(E)**, gram staining, Bar 20 μm]. AML, anterior mitral leaflet; LA, left atrium; PML, posterior mitral leaflet.

A 71-year-old man with fever and hematuria was admitted to our hospital because of urethral injury during Foley catheter replacement. At admission, physical examination results were as follows: blood pressure, 172/76 mmHg; high-grade fever, 39.0°C; tachycardia, 102 beats/min; and no remarkable cardiac murmur. He had a history of type 2 diabetes requiring insulin and a history of bilateral amputation for gangrene. He developed end-stage kidney failure and underwent hemodialysis twice weekly for 6 months previously. Simultaneously, he was still able to micturate with the aid of an indwelling urinary catheter. Chest radiography revealed cardiomegaly and bilateral pleural effusion. Laboratory test results revealed increased C-reactive protein levels (6.08 mg/dL, normal <0.3 mg/dL). Urine dipstick revealed glucose, blood 3+, protein 3+, and bacteria 3+. Urine microscopy revealed >100 white blood cells and >100 red blood cells/high-power field. Pelvic computed tomography revealed that the balloon of the Foley catheter was observed in the prostatic urethra, suggesting urethral injury (Supplementary Figure 1A). After conducting urine culture and two sets of blood cultures (BCs), intravenous minocycline (200 mg/day) was initiated as an empirical antimicrobial therapy for urinary tract infection (UTI). On day 4, follow-up laboratory test results revealed leukocytosis (16,600/μL) with further increased C-reactive protein levels of 15.9 mg/dL (Supplementary Figure 1B). Transthoracic echocardiography (TTE) revealed MAC involving the posterior mitral annulus, which was significantly more severe than that noted 1 year ago. Notably, an inhomogeneous mobile mass superimposed on the MAC was observed despite the absence of remarkable mitral regurgitation, suggesting vegetations (Figures 1A–C; Supplementary Videos 1, 2). Brain magnetic resonance imaging revealed multiple cerebral infarctions compatible with systemic embolism (Figures 1D–G). BCs and urine culture collected on admission yielded *Klebsiella pneumoniae*, which is susceptible to conventional antimicrobials but naturally resistant to ampicillin (Supplementary Table 1). These findings fulfilled the modified Duke criteria for the diagnosis of definite IE: 1 major and 3 minor criteria. Thus, the antimicrobial treatment was switched to intravenous ceftriaxone (4 g/day) for *K. pneumoniae* IE. However, because the fever continued, multiple separate BCs were performed on day 5 and thereafter. TEE further characterized the vegetations involving the MAC. 2D/3D TEE revealed a growing and prolapsing mass of vegetations originating from the MAC (Figures 2A–C; Supplementary Videos 3, 4). Nevertheless, intact valve leaflets and no significant mitral regurgitation were observed. The 4D Flexi-slice mode further clarified the morphological details of the MAC. Notably, the MAC where vegetations were attached was located around the P2 segment of the mitral valve (MV) and contained a hypoechoic region in the center, suggesting an annular abscess (Figure 2D). In addition, Q-analysis demonstrated that the hypoechoic region showed a low-intensity signal, which was different from both adjacent calcification and normal tissue and indicates a distinct property, supporting this notion (Figure 2E). Based on these findings, a tentative diagnosis of IE involving a calcified mitral annular abscess was made. On day 9, cardiac surgery was performed after confirming that the patient had no worsening of neurologic deficits. Visual inspection of the MV revealed a large mass of vegetations superimposed on the calcified posterior annulus of the MV (Figures 3A–C). However, the whole posterior leaflet, except for its basal end, was intact. During removal of vegetations, a small amount of pus was released from the interior of the MAC. Subsequent careful abscess debridement, reconstruction of the posterior atrioventricular groove using a bovine pericardial patch, and subsequent MV replacement with a prosthetic valve (Epic 29 mm, St. Jude Medical) were performed. Both the vegetation tissue and abscess contents were immediately sent for bacterial identification. Multiple separate BCs obtained on day 5 yielded methicillin-resistant *Staphylococcus aureus* (MRSA). Thus, in addition to ceftriaxone treatment, intravenous vancomycin (0.75 g/2 days) was combined. Finally, gram-positive bacterial infection was confirmed by pathological examination of the resected MV (Figures 3D,E). In addition, both the excised tissue and abscess content yielded MRSA, identical to those from the BCs collected on day 5 (Supplementary Table 1). Thus, the final diagnosis of MRSA IE was made, which fulfilled the modified Duke criteria for a diagnosis of definite IE with 2 major criteria. On day 19, de-escalation of vancomycin monotherapy was performed. Subsequent long-term intravenous vancomycin followed by oral sulfamethoxazole/trimethoprim (800 mg−160 mg/day) was administered. The post-operative course was uneventful, and the patient remained symptom-free during 1-year follow-up.

**Figure 4:**
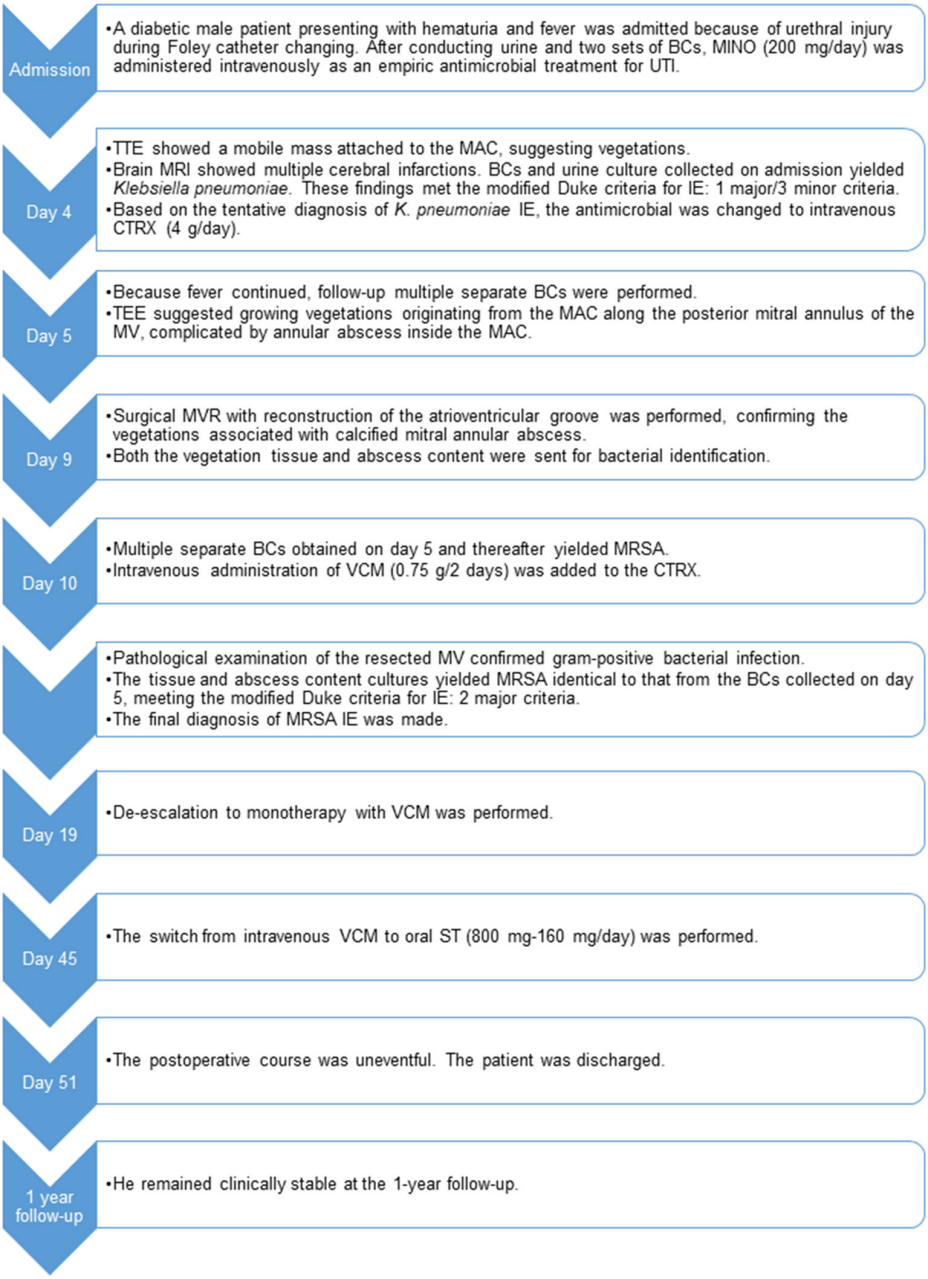
Timeline of case presentation. BCs, blood cultures; CTRX, ceftriaxone; IE, infective endocarditis; MAC, mitral annular calcification; MINO, minocycline; MRI, magnetic resonance imaging; MRSA, methicillin-resistant *Staphylococcus aureus*; MV, mitral valve; MVR, mitral valve replacement; ST, sulfamethoxazole/trimethoprim; TEE, transesophageal echocardiography; TTE, transthoracic echocardiography; UTI, urinary tract infection; VCM, vancomycin.

As a supplement, we present a timeline for the case presentation (Figure 4).

## Discussion

In general, MAC (characterized by progressive calcium deposition along the fibrous annulus of the MV) has a preponderance in women, and is more frequent/severe in older patients (Pomerance, 1970; Waller and Roberts, 1983; Benjamin et al., 1992; Massera et al., 2020). It was initially thought to be a passive degenerative process in the elderly, but it has recently been recognized to be a more active process because heart valve calcification shares many similarities with atherosclerosis (Johnson et al., 2006). MAC is subject to multiple contributory influences, including major risk factors of atherosclerosis, hemodynamic stress, chronic renal failure, abnormal mineral metabolism, and inflammation (Massera et al., 2020). MAC is associated with cardiovascular adverse events such as cardiogenic stroke, arrhythmia, or mitral valvular dysfunction (Benjamin et al., 1992; Massera et al., 2020). Moreover, MAC may be a predisposing factor for IE (Pressman et al., 2017). Herein, we present a rare case of dual infection with MAC-derived MRSA IE and *K. pneumoniae* UTI.

Our case has three implications. The first implication is that it underscores the limitations of the modified diagnostic Duke criteria for IE. The present case of IE showed a unique clinical course of dual bacteremia caused by two possible causative organisms (*K. pneumoniae* and MRSA), leading to a delayed diagnosis of MRSA IE. In fact, this case raises two difficulties in diagnosing MRSA IE. One difficulty is that MRSA was not identified in the first two sets of BCs collected on admission. Thus, a presumptive diagnosis of *K. pneumoniae* IE was made based on the initial BCs and echocardiographic findings, which met the modified Duke criteria for definite IE. The results of antimicrobial susceptibility testing of the MRSA identified in our case were that the organism was susceptible to erythromycin, clindamycin, and levofloxacin, strongly suggesting community-acquired MRSA (Naimi et al., 2003). Thus, it is highly likely that the patient had a dual infection of community-acquired MRSA IE and *K. pneumoniae* UTI on admission. In our case, presumed results of the initial BCs might have been transiently negative for MRSA because the viable MRSA was likely to have penetrated deep into the thrombus or MAC so that the surface remained sterile. Another difficulty is that both of the possible causative organisms were compatible with definitive IE based on clinical criteria. Although the modified Duke criteria for the diagnosis of IE are widely accepted, there are no clear indicators in the setting of dual bacteremia (Durack et al., 1994). Taking into account the patient's past medical history and the high virulence of the organisms, prompt, and appropriate antimicrobial therapy should be administered. In a prospective observational cohort study involving 1,779 cases of definite IE as classified by the Duke criteria among 16 western countries, *Staphylococcus aureus* (SA) was the dominant causative organism of IE, accounting for 31.6% of cases (Fowler et al., 2005). Notably, MRSA IE accounted for 33.3% of cases with SA IE, and significant factors predispose to the development of MRSA IE, including chronic hemodialysis, persistent bacteremia, chronic immunosuppressive treatment, health-care-associated infection, intravascular device source, or diabetes (Fowler et al., 2005). In contrast, the incidence of infections caused by non-HACEK gram-negative bacteria, including *K. pneumoniae*, is low, accounting for 2.1% (Fowler et al., 2005). The risk of IE is only 1.2% even in patients with *K. pneumoniae* bacteremia (Anderson and Janoff, 1998). Presumably, the poor ability of *K. pneumoniae* to adhere to cardiac valvular tissue compared to that of gram-positive organisms may be responsible for the low rate of IE (Gould et al., 1975). Our case involved a patient with diabetes undergoing hemodialysis with an indwelling urethral catheter. Considering the high virulence of MRSA, vancomycin should have been used at the initial presentation for suspected MRSA IE. Our case illustrates the importance of performing repeat BCs and careful clinical follow-up in cases of any rare causative organism, even when organism clinically meets the definite IE criteria.

The second implication is that our case highlights the importance of treating MAC-related IE as a malignant subtype of IE owing to the following three distinct features. First, unlike common leaflet endocarditis, MAC-related IE is rare, with a low incidence of 1.2% of all IE cases based on autopsy studies (Pomerance, 1970), and has the characteristic of developing even in the absence of pre-existing cardiac valvular dysfunction (Fernicola and Roberts, 1993). Based on the anatomical characteristics of the mitral annulus, MAC-related IE is invasive and easily spreads to surrounding tissues, with a high incidence of deadly periannular complications (e.g., abscess, perforation, fistula, ventricular pseudoaneurysm, hemopericardium, or purulent pericarditis) and systemic complications (e.g., embolic strokes, or neurologic events) (Eicher et al., 2004; Tsunekawa et al., 2006; Wentzell and Nair, 2018; Ozawa et al., 2019), leading to a poor prognosis with a high in-hospital mortality of 53% (Eicher et al., 2004). In addition, patients with significant mitral regurgitation caused by MAC-related IE often require extensive cardiac surgery with high postoperative mortality rates (in-hospital mortality, 29%; 3-year postoperative survival, only 49%) (Vistarini et al., 2007). Second, infection with MAC is more likely to occur in immunocompromised hosts, such as those with diabetes or malignancy (Eicher et al., 2004). In fact, common comorbidities might have had an impact on our patient's eventful postoperative course (Vistarini et al., 2007). Similarly, our case was an immunocompromised diabetic patient on hemodialysis, which would have been a predisposing risk factor for the occurrence of MAC-related IE, exemplifying the importance of timely treatment.

The third characteristic is that SA is the predominant causative microorganism of MAC-related IE, as in our case. In a study that retrospectively analyzed echocardiographic data from 56 patients with native MV IE, two major causative organisms were identified: SA and streptococci accounted for 50 and 30% of cases, respectively. SA was identified in 57% of cases of vegetations directly attached to MAC, whereas streptococci were exclusively identified in 94% of cases of vegetations attached to the leaflets, strongly suggesting that MAC can be a fertile medium for predominantly SA IE (Pressman et al., 2017). Although the exact mechanism of MAC remains poorly understood, bone formation, and remodeling are observed in calcified heart valves, which are presumably related to the differentiation of valve interstitial cells into osteoblast-like cells through an inflammatory process (Mohler et al., 2001; Rutkovskiy et al., 2017; Grim et al., 2020). SA is the most frequent organism in osteoarticular infections because SA can utilize a unique mechanism by which it can easily attach to, invade, and internalize into osteoblasts, thereby protecting itself from antimicrobial agents and host immune responses (Josse et al., 2015). Therefore, it is reasonable to predict that MAC can provide a rich nidus for SA infection.

The final implication is that 4D TEE was helpful for accurate diagnosis and proper surgical planning in our case. Differential diagnoses of echogenic round mass around the atrioventricular groove observed on TTE include MAC, abscess, caseous calcification of the mitral annulus, cyst, and tumor. Characteristic echocardiographic findings for MAC-related IE include “puffed-up” appearance indicating a swollen infected mitral annulus, or “stippled” appearance indicating focal calcium deposition within the vegetations (Eicher et al., 2004; Pressman et al., 2017). Similarly, in our patient, the unique fresh vegetations with a stippling observed on TEE enabled us to reach the correct diagnosis. Abscess formation is a prognostic factor independently associated with mortality in patients with native valve IE (odds ratio, 2.4; 95% confidence interval, 1.1–5.6) (Miro et al., 2005). Given the high complication rate of periannular abscesses in MAC-related IE, early diagnosis and prompt surgery are required (Eicher et al., 2004; Hill et al., 2007). While conventional TEE is generally more sensitive than TTE in detecting a periannular abscess, in the presence of MAC, especially involving the posterior mitral annulus, the ability to detect an abscess is significantly reduced even using TEE. In a TEE study that enrolled 115 patients with definite IE who underwent cardiac surgery, 44 patients had an abscess identified at the time of surgery (Hill et al., 2007). Less than half of these abscesses were detected by TEE preoperatively. The majority of missed abscesses (61%) were confined to the posterior region of the calcified mitral annulus, presumably due to artifacts and acoustic shadowing caused by MAC. However, 4D TEE can provide more detailed information about the location, internal properties, and possible complications of the MAC because the Flexi-slice mode allows us to slice in any direction. This approach was also effective in detecting the calcified annular abscess in this case, although further studies are needed to determine whether 4D TEE can identify any abscesses in patients with severe MAC.

## Conclusion

Herein, we report a unique case of dual infection with MRSA IE involving calcified mitral annular abscess and *K. pneumoniae* UTI, both of which met the modified Duke criteria for definite IE. The technique of 4D TEE is also valuable for corrective diagnosis and proper surgical planning. MAC-related IE is a rare, yet life-threatening, virulent subtype of IE. Therefore, early diagnosis and timely treatment are required. Clinicians should be aware of the clinical importance of this rare entity, and it is imperative that they do not hesitate to perform TEE for the accurate diagnosis and detection of its complications, and empirical antimicrobial therapy to treat MRSA.

## Data Availability Statement

The original contributions presented in the study are included in the article/Supplementary Materials, further inquiries can be directed to the corresponding author/s.

## Author Contributions

HYamam contributed to the clinical design and concept. HYamam, HYamad, KH, and TH acquired the clinical data. YI performed pathological analyses. HYamam and TT interpreted the data and drafted and revised the manuscript. All authors discussed, read, approved the manuscript, and authorized its submission for publication.

## Conflict of Interest

The authors declare that the research was conducted in the absence of any commercial or financial relationships that could be construed as a potential conflict of interest.

## Publisher's Note

All claims expressed in this article are solely those of the authors and do not necessarily represent those of their affiliated organizations, or those of the publisher, the editors and the reviewers. Any product that may be evaluated in this article, or claim that may be made by its manufacturer, is not guaranteed or endorsed by the publisher.

## Supplementary Material

Supplementary Figure 1 **(A)** Pelvic computed tomography (right, coronal view; left, axial view). Computed tomography reveals that the inflated balloon of a Foley catheter is inflated in the position of the prostatic urethra (arrow). **(B)** Timeline of laboratory values, fever, and, antimicrobial therapy. CRP, C-reactive protein; CTRX, ceftriaxone; MINO, minocycline; ST, sulfamethoxazole/trimethoprim; VCM, vancomycin; WBC, white blood cells.

Supplementary Table 1 Antimicrobial activities of antimicrobials against *Klebsiella pneumoniae* and *Staphylococcus aureus* isolated from blood and operation specimens in the present case. Identification of bacterial agents was performed by using an automated identification system based on the biochemical properties and/or matrix-assisted laser desorption ionization-time of flight mass spectrometry, in addition to the Gram-staining findings. The antimicrobial susceptibility phenotype (susceptible (S)/resistant (R)) was determined using disk diffusion or broth microdilution methods, according to the Clinical and Laboratory Standards Institute guidelines. MIC, minimum inhibitory concentration; ND, not determined.

Supplementary Video 1 Transthoracic echocardiography in apical four-chamber view. Note the echogenic round mass superimposed on the calcified posterior annulus of the mitral valve.

Supplementary Video 2 Color Doppler transthoracic echocardiography in parasternal long axis view. Note the absence of significant mitral regurgitation.

Supplementary Video 3 Three-dimensional transesophageal echocardiography. Note the prolapsing mass of the vegetations directly attached to the calcified posterior annulus of the mitral valve.

Supplementary Video 4 Two-dimensional color Doppler transesophageal echocardiography. Note the intact leaflets of the mitral valve, and absence of significant mitral regurgitation.

## Abbreviations

IE: infective endocarditis
MAC: mitral annular calcification
TEE: transesophageal echocardiography
BCs: blood cultures
UTI: urinary tract infection
TTE: transthoracic echocardiography
MV: mitral valve
MRSA: methicillin-resistant *Staphylococcus aureus*
SA: *Staphylococcus aureus*.
