# Supplementary figures and images for: Case Report: “Methicillin-Resistant Staphylococcus aureus Endocarditis Overlying Calcified Mitral Annular Abscess Misdiagnosed as Klebsiella pneumoniae Endocarditis”
Source: Front Microbiol. 2022 Jan 18;12:818219. doi: 10.3389/fmicb.2021.818219 (PMC8804532; doi:10.3389/fmicb.2021.818219)

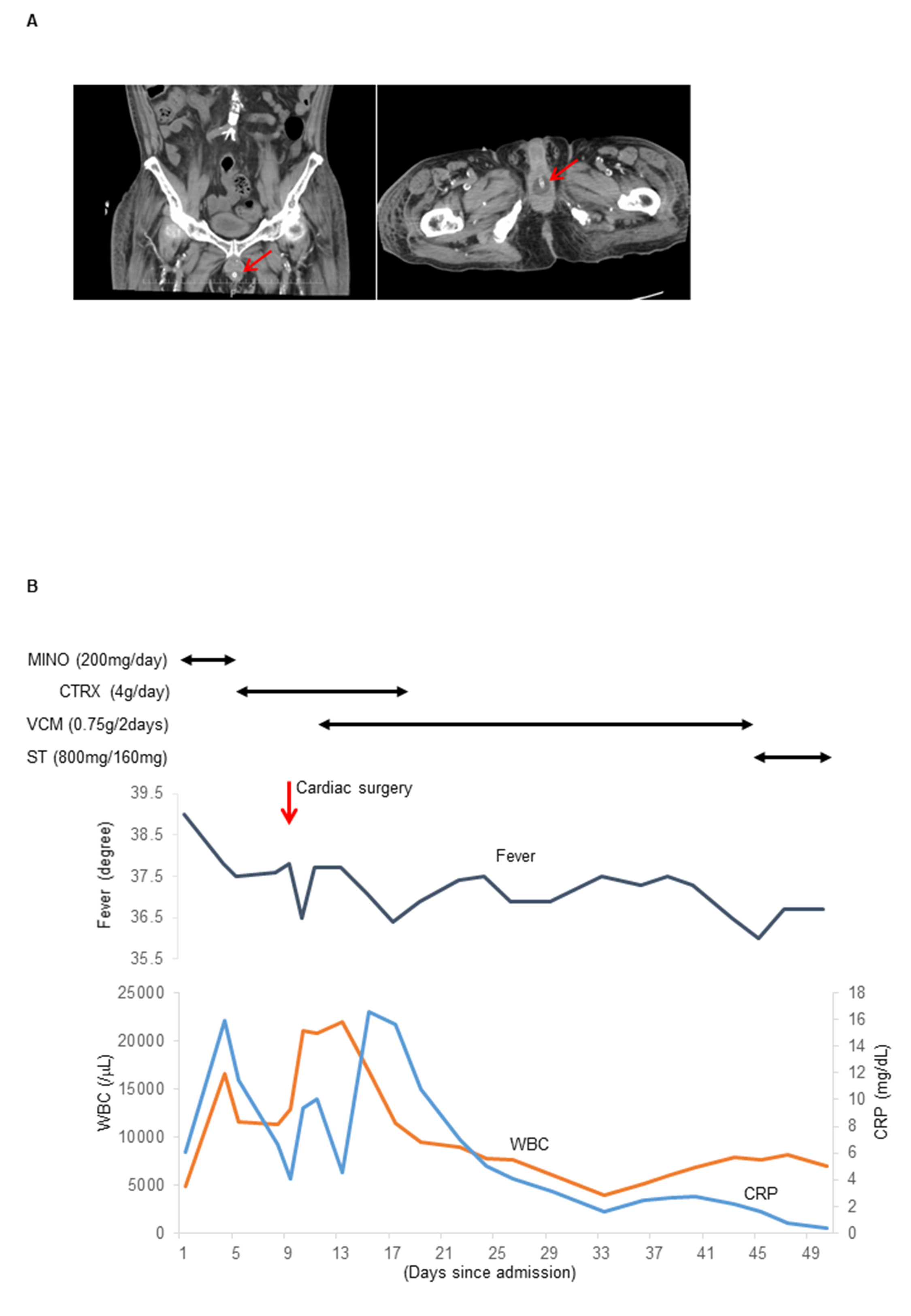

Supplement: Supplementary Figure 1 — (A) Pelvic computed tomography (right, coronal view; left, axial view). Computed tomography reveals that the inflated balloon of a Foley catheter is inflated in the position of the prostatic urethra (arrow). (B) Timeline of laboratory values, fever, and, antimicrobial therapy. CRP, C-reactive protein; CTRX, ceftriaxone; MINO, minocycline; ST, sulfamethoxazole/trimethoprim; VCM, vancomycin; WBC, white blood cells. [file Image_1.TIF]
