# Supplementary material for: Case Report: “Methicillin-Resistant Staphylococcus aureus Endocarditis Overlying Calcified Mitral Annular Abscess Misdiagnosed as Klebsiella pneumoniae Endocarditis”
Source: Front Microbiol. 2022 Jan 18;12:818219. doi: 10.3389/fmicb.2021.818219 (PMC8804532; doi:10.3389/fmicb.2021.818219)
Supplement: Supplementary file 7 [file Data_Sheet_1.PDF]

CARE case report guidelines

CARE Checklist of information to include when writing a case report

(cc) BY-NC-ND

Topic	Item	Checklist item description	Reported on Line
Title	1	The diagnosis or intervention of primary focus followed by the words "case report"	2-3
Key Words	2	2 to 5 key words that identify diagnoses or interventions in this case report, including "case report"	22-23
Abstract (no references)	3a	Introduction: What is unique about this case and what does it add to the scientific literature?	29-33
	3b	Main symptoms and/or important clinical findings	34-35
	3c	The main diagnoses, therapeutic interventions, and outcomes	36-38
	3d	Conclusion—What is the main "take-away" lesson(s) from this case?	39-41
Introduction	4	One or two paragraphs summarizing why this case is unique (**may include references**)	49-53
Patient Information	5a	De-identified patient specific information.	56
	5b	Primary concerns and symptoms of the patient.	56-57
	5c	Medical, family, and psycho-social history including relevant genetic information	59-62
	5d	Relevant past interventions with outcomes	N/A
Clinical Findings	6	Describe significant physical examination (PE) and important clinical findings.	57-59
Timeline	7	Historical and current information from this episode of care organized as a timeline	N/A
Diagnostic Assessment	8a	Diagnostic testing (such as PE, laboratory testing, imaging, surveys).	62-67, 69-79, 81-89, 100-104
	8b	Diagnostic challenges (such as access to testing, financial, or cultural)	78-80
	8c	Diagnosis (including other diagnoses considered)	67-69, 79-80, 90-91, 103
	8d	Prognosis (such as staging in oncology) where applicable	N/A
Therapeutic Intervention	9a	Types of therapeutic intervention (such as pharmacologic, surgical, preventive, self-care)	99-100
	9b	Administration of therapeutic intervention (such as dosage, strength, duration)	68-69
	9c	Changes in therapeutic intervention (with rationale)	78-80, 100, 104-107
Follow-up and Outcomes	10a	Clinician and patient-assessed outcomes (if available)	107-108
	10b	Important follow-up diagnostic and other test results	N/A
	10c	Intervention adherence and tolerability (How was this assessed?)	N/A
	10d	Adverse and unanticipated events	N/A
Discussion	11a	A scientific discussion of the strengths AND limitations associated with this case report	123-208
	11b	Discussion of the relevant medical literature **with references**.	112-208
	11c	The scientific rationale for any conclusions (including assessment of possible causes)	112-208
	11d	The primary "take-away" lessons of this case report (without references) in a one paragraph conclusion	211-217
Patient Perspective	12	The patient should share their perspective in one to two paragraphs on the treatment(s) they received	N/A
Informed Consent	13	Did the patient give informed consent? Please provide if requested	Yes ☑ No ☐
